# Exogenous Gamma-Aminobutyric Acid Application Induced Modulations in the Performance of Aromatic Rice Under Lead Toxicity

**DOI:** 10.3389/fpls.2022.933694

**Published:** 2022-07-22

**Authors:** Umair Ashraf, Sammina Mahmood, Shakeel Ahmad Anjum, Rana Nadeem Abbas, Fahd Rasul, Javed Iqbal, Zhaowen Mo, Xiangru Tang

**Affiliations:** ^1^Department of Botany, Division of Science and Technology, University of Education, Lahore, Pakistan; ^2^Department of Crop Science and Technology, College of Agriculture, South China Agricultural University, Guangzhou, China; ^3^Department of Agronomy, University of Agriculture, Faisalabad, Pakistan; ^4^Department of Agricultural Engineering, Khawaja Fareed University of Engineering and Information Technology, Rahim Yar Khan, Pakistan; ^5^State Key Laboratory for Conservation and Utilization of Subtropical Agro-bioresources, College of Agriculture, South China Agricultural University, Guangzhou, China

**Keywords:** antioxidants, GABA, lead, photosynthesis, rice

## Abstract

Gamma-aminobutyric acid (GABA) is a non-protein amino acid and has a multi-functional role in abiotic stress tolerance. A pot experiment was conducted to assess the role of exogenous gamma-aminobutyric acid (GABA) application to modulate the growth, yield, and related physio-biochemical mechanisms in two aromatic rice cultivars, that is, Guixiangzhan (GXZ) and Nongxiang 18 (NX-18), under Pb toxic and normal conditions. The experimental treatments were comprised of Ck: without Pb and GABA (control), GABA: 1 mM GABA is applied under normal conditions (without Pb), Pb + GABA: 1 mM GABA is applied under Pb toxicity (800 mg kg^−1^ of soil), and Pb= only Pb (800 mg kg^−1^ of soil) is applied (no GABA). The required concentrations of GABA were applied as a foliar spray. Results revealed that Pb stress induced oxidative damage in terms of enhanced malondialdehyde (MDA), electrolyte leakage (EL), and H_2_O_2_ contents, while exogenous GABA application improved leaf chlorophyll, proline, protein and GABA contents, photosynthesis and gas exchange, and antioxidant defense under Pb toxicity in both rice cultivars. Moreover, glutamine synthetase (GS) and nitrate reductase (NR) activities were variably affected due to GABA application under Pb stress. The yield and related traits, that is, productive tillers/pot, grains/panicle, filled grain %, 1,000-grain weight, and grain yield were 13.64 and 10.29, 0.37% and 2.26%, 3.89 and 19.06%, 7.35 and 12.84%, and 17.92 and 40.56 lower under Pb treatment than Pb + GABA for GXZ and NX-18, respectively. Furthermore, exogenous GABA application in rice reduced Pb contents in shoot, leaves, panicle, and grains compared with Pb-exposed plants without GABA. Overall, GXZ performed better than NX-18 under Pb toxic conditions.

## Introduction

Environmental pollutants are well known due to their toxic effects on plants and the extent of these effects is generally dependent on their concentration, persistence, speciation, and toxicity potential (Ashraf et al., 2020; Aslam et al., 2021; Huang et al., 2021). Pollutants commonly enter into the plant systems *via* roots (from the soil) or direct depositions externally from the open environment Soil and/or air contamination with such pollutants are due to continued usage and waste emission from industries, contaminated wastewater or sewage sludge application to agricultural lands, mining activities, wide use of automobiles, and chemical usage in agriculture (Farid et al., 2020). Lead (Pb) is known as the most potent pollutant (Tian et al., 2014; Khan et al., 2016) and its eco-toxicological manifestations for plants are widely reported in previous literature (Cheng and Hu, 2010; Ashraf et al., 2015, 2017a). According to the (United States Geological Survey, 2009), the highest recoverable Pb from mining was generated by China followed by Australia and USA with 1,690, 516, and 400 thousand metric tons of annual production.

Lead (Pb) often has serious consequences for plants from morpho-physiological and genetic levels to growth, yield, and quality produce (Guedes et al., 2021). For example, Pb inhibits root/shoot growth and causes photosynthetic capacities, chloroplastic ultra-structures, chlorophyll biosynthesis, nutritional imbalance, and yield and quality traits (Maestri et al., 2010; Ali et al., 2014b,c; Ashraf and Tang, 2017). The presence of Pb in soil and its subsequent uptake by plants is substantially affected by plant and soil factors as well as forms of Pb in soil solution (Tangahu et al., 2011; Ashraf et al., 2015); however, the availability of free Pb^2+^ ions in the soil solution is often assessed by the participation of Pb in adsorption/desorption processes within the soil (Vega et al., 2010). Plant roots are the first organs that come into contact with soil Pb, which then transfers to above-ground plant parts and damages the physio-biochemical mechanisms as well as yield and food quality deterioration in plants (Uzu et al., 2010; Ashraf et al., 2020; Ma et al., 2021).

Rice is a major cereal crop in many Asian countries (Abid et al., 2015), of which fragrant rice is well-famous owing to have its special aroma and excellent cooking qualities (Singh et al., 2000; Bryant and McClung, 2011; Li M. et al., 2016); however, soil contamination with toxic heavy metals especially Pb may cause substantial reduction in growth and productivity of aromatic rice.

Gamma-aminobutyric acid (GABA) is often involved in signaling transduction mechanisms in plants under different abiotic stresses such as chilling, heat, osmotic, respiratory stress, and so on (Yang et al., 2011; Vergara et al., 2013; Nayyar et al., 2014; Vijayakumari and Puthur, 2016), as well as modulates numerous physio-biochemical responses that help plants to cope against these stresses. GABA-induced signaling against abiotic stresses is also associated with the activation of the antioxidative defense system, osmoregulation, pH maintenance, and buffering tool for C and N metabolism (Bouche and Fromm, 2004; Li W. et al., 2016). To the best of our knowledge, the GABA-induced regulations in physio-biochemical processes and yield of aromatic rice under Pb toxicity have never been studied. Therefore, the current study was conducted to assess the GABA-induced modulations in the performance of aromatic rice under normal and Pb toxic conditions.

## Materials and Methods

### Experimental Setup

A pot experiment was conducted in a rain-protected net house at Experimental Research Area, College of Agriculture, South China Agricultural University, Guangzhou, China (23°09′ N, 113°22′ E, and 11 m above the sea level) from April to July 2016. Seeds of two aromatic rice cultivars, that is, Guixiangzhan (GXZ) and Nongxiang-18 (NX-18), were obtained from the College of Agriculture, South China Agricultural University, Guangzhou, China. The seeds were surface sterilized with NaClO_4_ (5% sol.) for 10 min, rinsed with distilled water, and then dipped in tap water overnight to germinate. The germinated seeds were then sown in soil-filled plastic trays (specially designed for nursery raising), placed on a leveled field, and carefully covered with a plastic sheet. On the other hand, the plastic pots (height: 25 cm; diameter: 32 cm) were filled with 10 kg of air-dried soil having 79–80 mg kg^−1^ available N, 9.0–9.5 mg kg^−1^ available P, and 120–125 mg kg^−1^ available K, 20–22 g kg^−1^ organic matter, 5.58 soil pH (moderately acidic), and 45 ± 5 mg kg^−1^ soil Pb contents. Before transplanting, the Pb(NO_3_)_2_ salt was added in solution form into the soil at the required concentration (800 mg kg^−1^ of soil) and mixed (Ashraf and Tang, 2017) 10 days before transplanting. The pots were applied with water with 3–4 cm water layer above the soil till transplantation of rice seedlings. Uniformly grown and well-developed 25 days old rice seedlings were transplanted into the pots with five hills per pot and three to four seedlings per hill. The experimental treatments were comprised of Ck: without Pb and GABA (control), GABA: 1 mM GABA is applied under control conditions (without Pb), Pb + GABA: 1 mM GABA is applied under Pb toxicity (800 mg kg^−1^ of soil), and Pb= only Pb (800 mg kg^−1^ of soil) is applied (no GABA). The GABA at 1 mM was exogenously applied uniformly at the active tillering stage (3 weeks after transplanting) till the runoff through leaves. The optimized GABA levels are based on previous studies by Nayyar et al. (2014) who used a range of GABA concentrations (0.25–2 mM) and found 1 mM as the most suitable one for rice under stress conditions. Moreover, the NPK was applied at 2.30, 3.50, and 1.50 g to all pots, and water was applied regularly to keep the soil saturated with water.

### Sampling and Data Collection

Plant leaves were sampled randomly at 7, 14, and 21 days after treatment (DAT) and stored at −80°C for plant physio-biochemical characters and chlorophyll contents, whereas for Pb quantification, the plants were sampled at late vegetative (VEG), panicle heading (PH) and maturity (MAT) stages, oven-dried, ground into powder form, and stored for digestion purpose.

### Biochemical Assays

The contents of hydrogen peroxide (H_2_O_2_) were assessed according to Velikova et al. (2000) and the final H_2_O_2_ contents were denoted as μmol g^−1^ fresh weight (FW). The contents of malondialdehyde (MDA) were determined according to (Hodges et al., 1999) and the final contents were presented as μmol g^−1^ FW with the formula:


(1) $$\begin{array}{l} \text{MDA Contents }(\text{umol }{\text{g}}^{-\text{1}}\text{ FW})=\{645\left(OD532-600\right)-\left(0.56OD450\right)\} \end{array}$$

Electrolyte leakage (EL) was determined according to (Valentovic et al., 2006):


(2) $$\begin{array}{l} \left(\%\right)=\;\frac{EC1}{EC2}\;\times 10EL\;0 \end{array}$$

Leaf chlorophyll contents, that is, Chl a, Chl b, and carotenoids were estimated according to (Arnon, 1949) and the absorbance was recorded at 665, 649, and 470 nm. Leaf proline contents were estimated according to Bates et al. (1973) by using ninhydrin whereas the protein contents in leaves were estimated according to the methods advised by (Bradford, 1976) using G-250. The GABA contents in leaves were estimated according to Zhao et al. (2009). In brief, fresh leaves (0.2 g) were homogenized in 5 mL of 60% ethanol and then put in an oscillator (HZS-H, China) for 4 h at 200 oscillations min^−1^, centrifuged at 8,000 rpm for 5 min, and 1 ml of it was mixed with 0.6 ml of 0.2 mol l^−1^ sodium tetraborate, 2 ml of 5% phenol, and 1 ml of 7% sodium hypochlorite, and heated at 100°C and then cooled down in water bath. The absorption of the reaction mixture was read at 645 and the GABA contents were determined and expressed as μg g^−1^ FW.

Fresh leaves samples (0.3 g) were homogenized in 6 ml of 50 mM sodium phosphate buffer (pH 7.8) with pestle and mortar and the homogenate was centrifuged at 8,000 rpm for 20 min at 4 C. The supernatant was then used to estimate the antioxidant enzyme activities.

Superoxide dismutase (SOD, EC 1.15.1.1) was determined according to Zhang et al. (2008) by following the inhibition of photochemical reduction due to nitro blue tetrazolium (NBT). The SOD activity per unit was the amount of enzyme required to inhibit NBT photochemical reduction to 50% as an activity unit (U). The activity of peroxidase (POD, EC 1.11.1.7) was determined according to (Zhang, 1992) whereas one unit of POD activity was the amount of enzyme that caused the decomposition of 1 μg substrate at 470 nm. Catalase activity (CAT, EC 1.11.1.6) was determined according to the protocols of Aebi (1984), whereas one unit of enzyme activity (U) was the decomposition of 1 M H_2_O_2_ at A_240_ within 1 min in 1 g of fresh leaves samples. Ascorbate peroxidase (APX, EC 1.11.1.11) activity and reduced glutathione (GSH) contents were estimated by using kits purchased from Nanjing Jiancheng Bioengineering Institute, China.

Glutathione synthetase (GS) activity was determined according to (Li, 2006). The fresh leaves (0.2 g) were homogenized in 3 ml of Tris-HCI (pH 8.0) extraction buffer containing 1.5295 g Tris, 0.1245 g MgSO_4‘_6H_2_O, 0.1543 g DTT, and 34.25 g sucrose in 250 ml water. The homogenate was centrifuged at 10,000 rpm for 20 min, and 0.7 ml of supernatant was added to 1.6 ml of assay, a solution mixture containing 3.0590 g Tris, 4.9795 g MgSO_4_.7H_2_O, 0.8628 g glutamic acid-Na, 0.6057 g cysteine, 0.1920 g amino polycarboxylic acid, and 1.3898 g hydroxylamine hydrochloride in 250 ml water and 0.7 ml ATP (0.1210 g ATP) in 5 ml water. The mixture was incubated for 30 min at 25°C. Subsequently, the reaction was stopped after adding 1 ml of color reagents [3.3176 g trichloro acetic acid (TCA), 10.1021 g acidic FeCl_3_.6H_2_O dissolved in water, and added 5 ml HCl, reached to 100 ml]. The samples were centrifuged at 4,000 rpm for 15 min, and the absorbance was read at 540 nm. The blank group contained 1.6 ml of an assay mixture containing 3.0590 g Tris, 4.9795 g MgSO_4_.7H_2_O, 0.8628 g glutamic acid-Na, 0.6057 g cysteine, and 0.1920 g aminopolycarboxylic acid. The GS activity was measured as: change in absorption (ΔA × Vt)/(Vs × FW × t), where ΔA = Change in absorbance; Vt = total volume; Vs = sample volume; FW = fresh weight; and t = reaction time.

Nitrate reductase (NR) activity was determined according to Yu and Zhang (2012) with some modifications. Fresh leaves (0.25 g) were precisely homogenized with 4.0 ml extraction buffer containing 0.1211 g cysteine and 0.0372 g EDTA-Na_2_ in 100 ml phosphate buffer (pH 7.5). The homogenate was clarified by centrifugation at 4,000 rpm for 15 min at 4°C. The supernatant was collected and assayed. Crude enzyme (0.4 ml) extract was added 1.2 ml KNO_3_ buffer (8.8640 g Na_2_HPO_4_.12H_2_O and 0.0570 g K_2_HPO_4_.3H_2_O in 1,000 ml water, pH 8.7) and 0.4 ml NADH [2 mg NADH in 1 ml phosphate buffer (pH 7.5)]. The mixture was kept for 30 min at 25°C. For control, 0.4 ml phosphate buffer was used, whereas the reaction was stopped after adding 1.0 ml of 1% each of 4-aminobenzene sulfonic acid and 0.2% 1-naphthylamine and left for 30 min for color development at 30°C. The tubes were then centrifuged at 4,000 rpm for 10 min and the absorbance was noted at 540 nm immediately. NR activity is expressed in Units h^−1^ g^−1^ fresh weight (FW), which refers to the amount of enzyme required to produce NO_2_ in 1 h by 1 g of FW.

### Photosynthesis and Gas Exchange

Net photosynthetic rate (A) and gas exchange attributes were determined in flag leaves of four plants from each treatment at the vegetative stage by using a portable photosynthesis system (LI-6400, LI-COR, USA) at 09:00–11:30 am according to (Pan et al., 2016).

### Determination of Pb Contents

Plant samples in powder form (0.2 g) of each part were digested with HNO_3_:HClO_4_ (4:1 *v/v*) on an electric digestion system and the resultant solutions were diluted to 25 ml and filtered. The Pb contents in different plant parts were estimated by using Atomic Absorption Spectrophotometer (AA6300C, Shimadzu, Japan).

### Determination of Yield and Related Attributes

Tillers bearing panicles were counted in each pot to get productive tillers per pot. Rice panicles of both rice cultivars were threshed manually to estimate the grains per panicle and filled grain percentage. At maturity, all remaining pots were harvested with sickle and manually threshed and the grains were sun-dried. Grain samples were taken randomly from a filled seed lot and weighed to get 1,000-grain weight. Grain yield pot^−1^ was the total grain weight from each pot.

### Experimental Design and Statistical Analysis

Pots were arranged in a completely randomized design with 10 pots per treatment, whereas the dataset was analyzed by “Statistix 8” (Analytical Software, Tallahassee, Florida, USA) while the least significant difference (LSD) (P_0.05_) was used to calculate the difference among treatment means. SigmaPlot 9.0 (Systat Software Inc., San Jose, CA, USA) was used to generate graphs.

## Results

### Oxidative Damage

**Figure 1:**
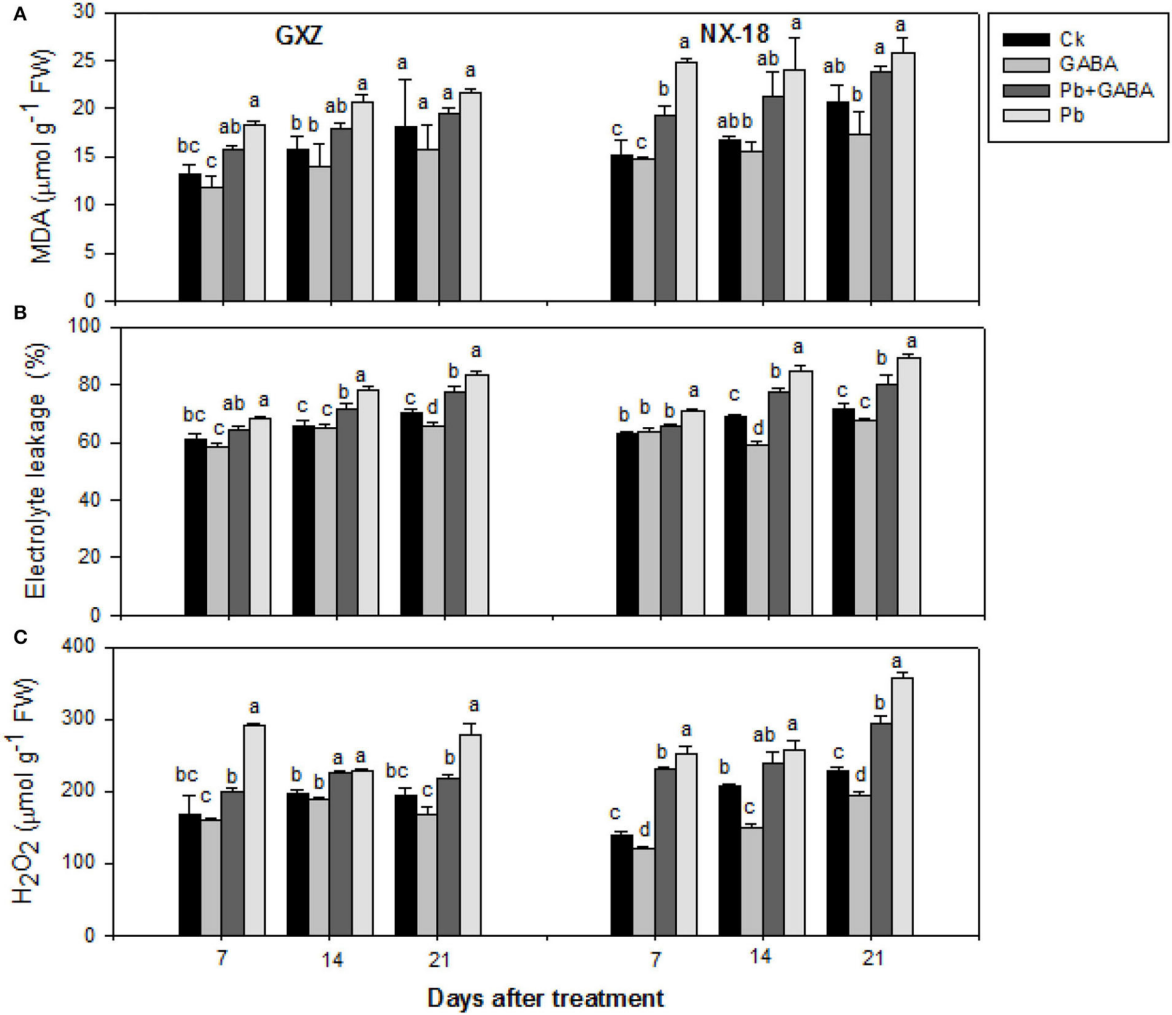
Influence of exogenous GABA on **(A)** malondialdehyde (MDA), **(B)** electrolyte leakage (EL), and **(C)** hydrogen peroxide (H_2_O_2_) contents in fragrant rice under Pb toxicity at 7, 14, and 21 days after treatment. Vertical bars are means ± S.E. Vertical bars sharing a letter in common do not differ significantly at *P* < 0.05. Ck: without Pb and GABA (control); GABA: 1 mM GABA; Pb + GABA: 1 mM GABA + 800 mg of Pb kg^−1^ of soil; Pb = 800 mg kg^−1^ of soil. GXZ, Guixiangzhan; NX-18, Nongxiang-18.

Pb stress enhanced MDA, EL, and H_2_O_2_ contents in both rice cultivars while exogenous GABA application protects against oxidative stress. Compared with Ck, the MDA contents were 39.64, 31.13, and 19.82% higher in GXZ and 63.44, 43.25, and 24.94% higher in NX-18 at 7, 14, and 21 DAT, respectively. The lowest MDA contents were found in GABA-treated rice plants without Pb followed by GABA + Pb. GABA also protected membrane permeability (reduced EL values) in both rice cultivars but the values were higher for NX-18 than GXZ. The EL values in Pb were almost 2-fold than Pb + GABA in both rice cultivars at 7, 14, and 21 DAT. Similarly, GABA was also found effective against H_2_O_2_ production in both rice cultivars. The H_2_O_2_ contents in Pb + GABA treatment were 46.39, 1.35, and 5.82% lower for GXZ as well as 9.65, 8.04, and 21.06% lower for NX-18 than only Pb treatment at 7, 14, and 21 DAT, respectively. Overall, the degree of oxidative damage was comparatively higher in NX-18 than in GXZ (Figure 1).

### Chlorophyll Contents and Carotenoids

**Figure 2:**
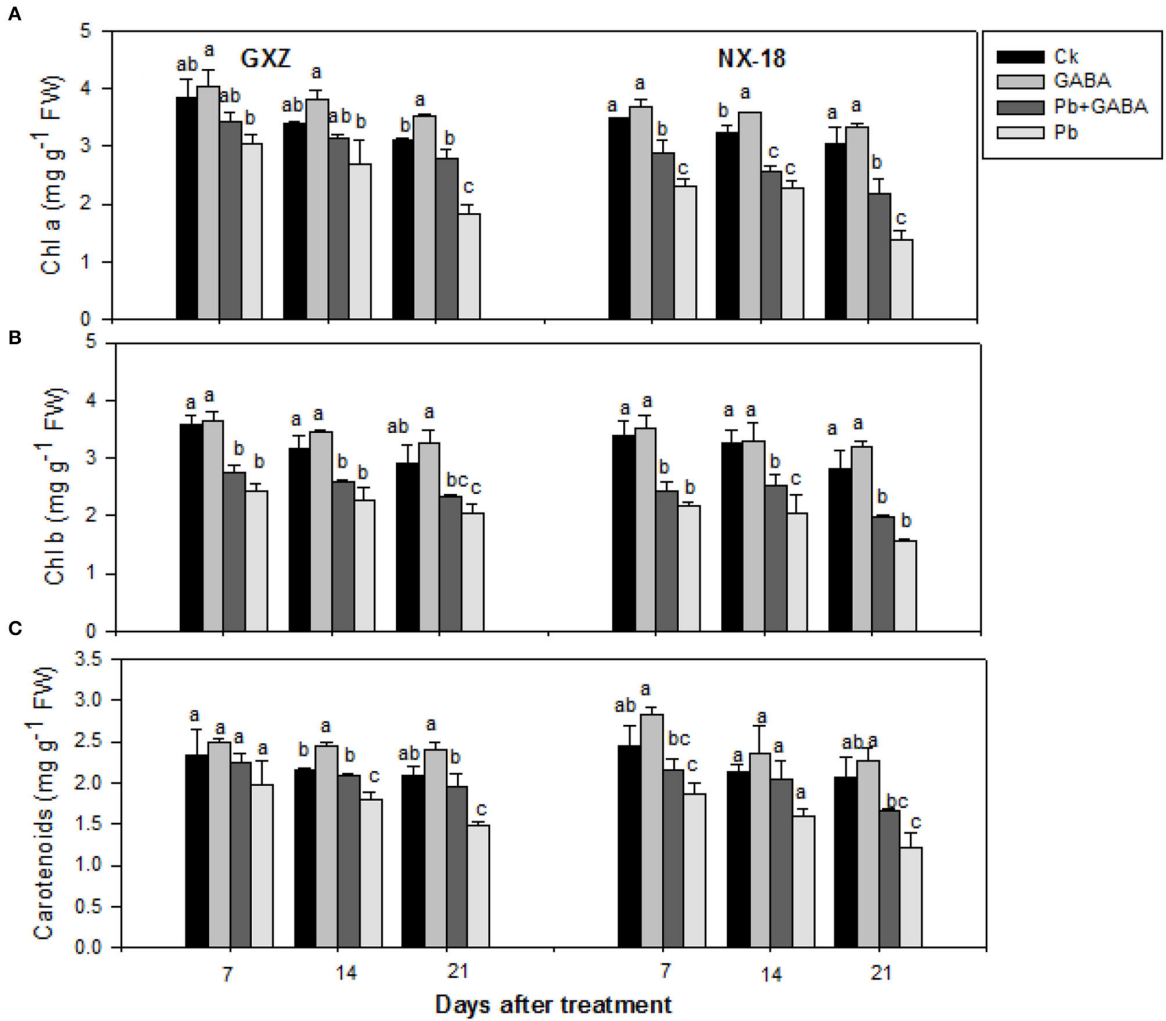
Influence of exogenous GABA on **(A)** chlorophyll a (Chl a), **(B)** chlorophyll b (Chl b), and **(C)** carotenoids (Car) contents in fragrant rice under Pb toxicity at 7, 14, and 21 days after treatment. Vertical bars are means ± S.E. Vertical bars sharing a letter in common do not differ significantly at *P* < 0.05. Ck: without Pb and GABA (control); GABA: 1 mM GABA; Pb + GABA: 1 mM GABA + 800 mg of Pb kg^−1^ of soil; Pb = 800 mg kg^−1^ of soil. GXZ, Guixiangzhan; NX-18, Nongxiang-18.

Significant reductions (*P* < 0.05) were noted in Chl a, Chl b, and carotenoids in both cultivars under Pb toxicity while exogenous GABA application was found effective to confer inhibitory effect of Pb on leaf chlorophyll contents. There was 21.28, 20.67, and 41.44% reduction in Chl a, 32.45, 28.55, and 29.45% in Chl b, and 15.82, 16.92, and 28.97% in carotenoids in GXZ and 33.80, 29.31, and 54.29% reduction in Chl a, 36.06, 37.18, and 44.09% in Chl b, and 23.65, 24.89, and 41.11% in carotenoids, which were recorded in NX-18 at 7, 14, and 21 DAT, respectively. Although Pb inhibited photosynthetic pigments in both rice cultivars, the foliar GABA application improved photosynthetic pigments in both rice cultivars. Maximum Chl a, Chl b, and carotenoids were recorded in GABA-treatment followed by Ck and Pb + GABA while least in Pb-treatment. In addition, the carotenoids were reduced but remained statistically similar (*P* > 0.05) in GXZ at 7 DAT under all treatments (Figure 2).

### Proline, Protein, and GABA Contents

**Figure 3:**
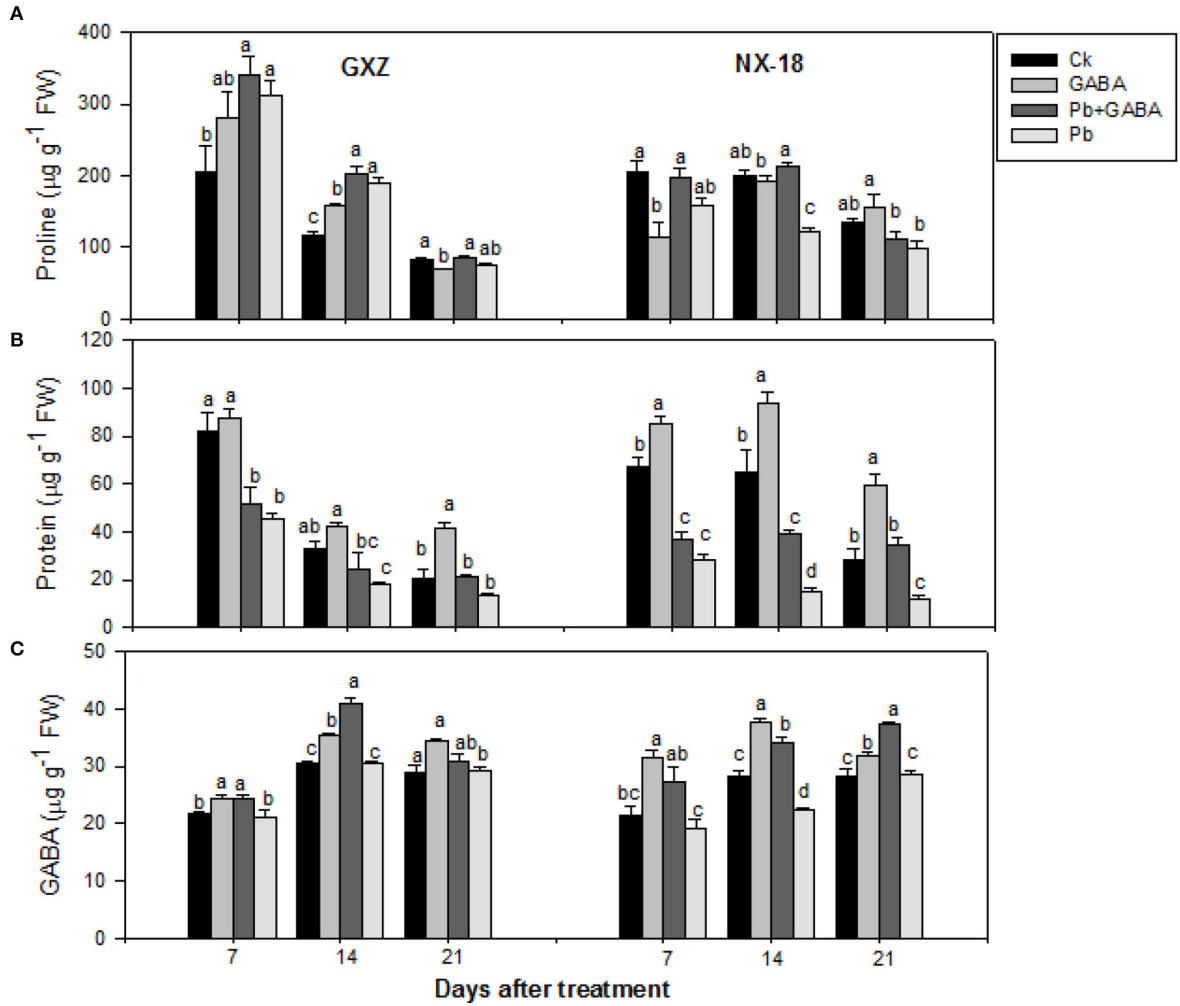
Influence of exogenous GABA on **(A)** proline, **(B)** protein, and **(C)** GABA contents in fragrant rice under Pb toxicity at 7, 14, and 21 days after treatment. Vertical bars are means ± S.E. Vertical bars sharing a letter in common do not differ significantly at *P* < 0.05. Ck: without Pb and GABA (control); GABA: 1 mM GABA; Pb + GABA: 1 mM GABA + 800 mg of Pb kg^−1^ of soil; Pb = 800 mg kg^−1^ of soil. GXZ, Guixiangzhan; NX-18, Nongxiang-18.

Significant improvements (*P* < 0.05) were noticed in leaf proline, protein, and GABA contents in both GXZ and NX-18 under GABA application, while Pb stress reduced protein contents dramatically. Maximum proline contents were recorded in Pb + GABA treatment which was 67.04, 74.18, and 1.41% higher than Ck at 7, 14, and 21 DAT, respectively, in GXZ, whereas proline contents in NX-18 were remained statistically similar (*P* > 0.05) for Ck and Pb + GABA at 7 and 14 DAT but Ck and only GABA at 21 DAT, respectively. In contrast, exposure to Pb severely reduced protein contents in both rice cultivars; however, the effects were more apparent in NX-18 than GXZ. In both rice cultivars, the maximum protein contents were recorded in GABA treatment (without Pb) followed by Ck, Pb + GABA, and Pb. Compared with Ck, the GABA application enhanced protein contents by 6.15, 26.19, and 102.80% in GXZ and 27.23, 44.50, and 91.10% in NX-18 at 7, 14, and 21 DAT, respectively. Compared with Ck, the values of the percentage decrease in protein contents were higher in NX-18 (57.47, 76.97, and 56.90%) than in GXZ (44.52, 44.40, and 35.01%). In addition, exogenous GABA application substantially improved leaves GABA contents. The highest GABA contents, that is, 24.43, 40.88, and 34.34 μg g^−1^, were noted in GABA, Pb + GABA, and GABA treatment in GXZ, 31.37 and 37.71 μg g^−1^ under GABA treatment, and 37.41 μg g^−1^ under Pb + GABA in NX-18 at 7, 14, and 21 DAT, respectively. Moreover, the lowest GABA contents were noticed under Pb treatment without GABA application in both rice cultivars (Figure 3).

### Net Photosynthesis and Gas Exchange Attributes

**Figure 4:**
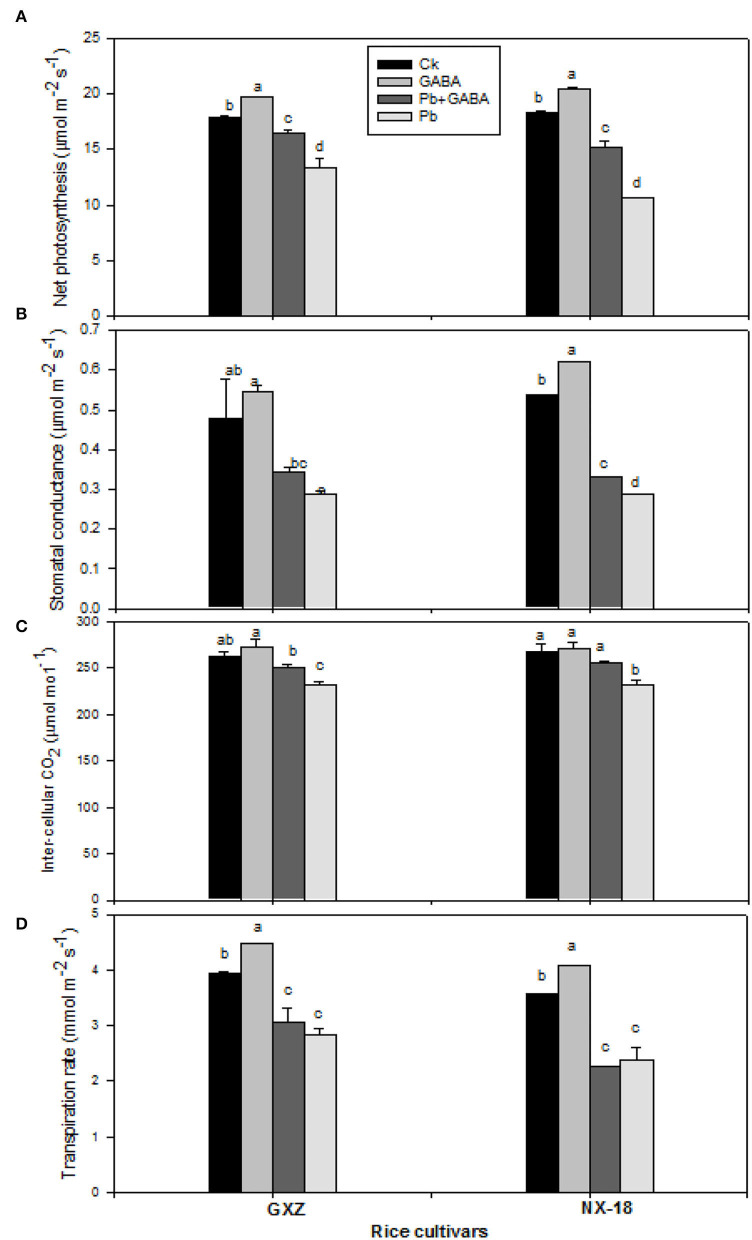
Influence of exogenous GABA on **(A)** net photosynthesis, **(B)** stomatal conductance, **(C)** inter-cellular CO_2_, and **(D)** transpirational rate in fragrant rice under Pb toxicity at 7, 14, and 21 days after treatment. Vertical bars are means ± S.E. Vertical bars sharing a letter in common do not differ significantly at *P* < 0.05. Ck: without Pb and GABA (control); GABA: 1 mM GABA; Pb + GABA: 1 mM GABA + 800 mg of Pb kg^−1^ of soil; Pb = 800 mg kg^−1^ of soil. GXZ, Guixiangzhan; NX-18, Nongxiang-18.

Pb toxicity led to a significant reduction in net photosynthesis, stomatal conductance, intercellular CO_2_, and transpiration rate against Ck; however, exogenous GABA application improved net photosynthesis and gas exchange attributes significantly. The GABA application enhanced net photosynthesis (9.86 and 11.18%), stomatal conductance (13.81 and 15.51%), intercellular CO_2_ (4.04 and 0.76%), and transpiration rate (13.41 and 3.58%) than Ck in GXZ and NX-18, respectively. Moreover, the GABA application under Pb treatment (GABA + Pb) enhanced the net photosynthesis by 19.46 and 29.93%, stomatal conductance by 15.74 and 13.27%, and intercellular CO_2_ by 7.69 and 9.23% for both GXZ and NX-18, respectively, and transpiration rate 7.92% (in GXZ only) as compared with Pb (Figure 4).

### Antioxidative Defense

**Figure 5:**
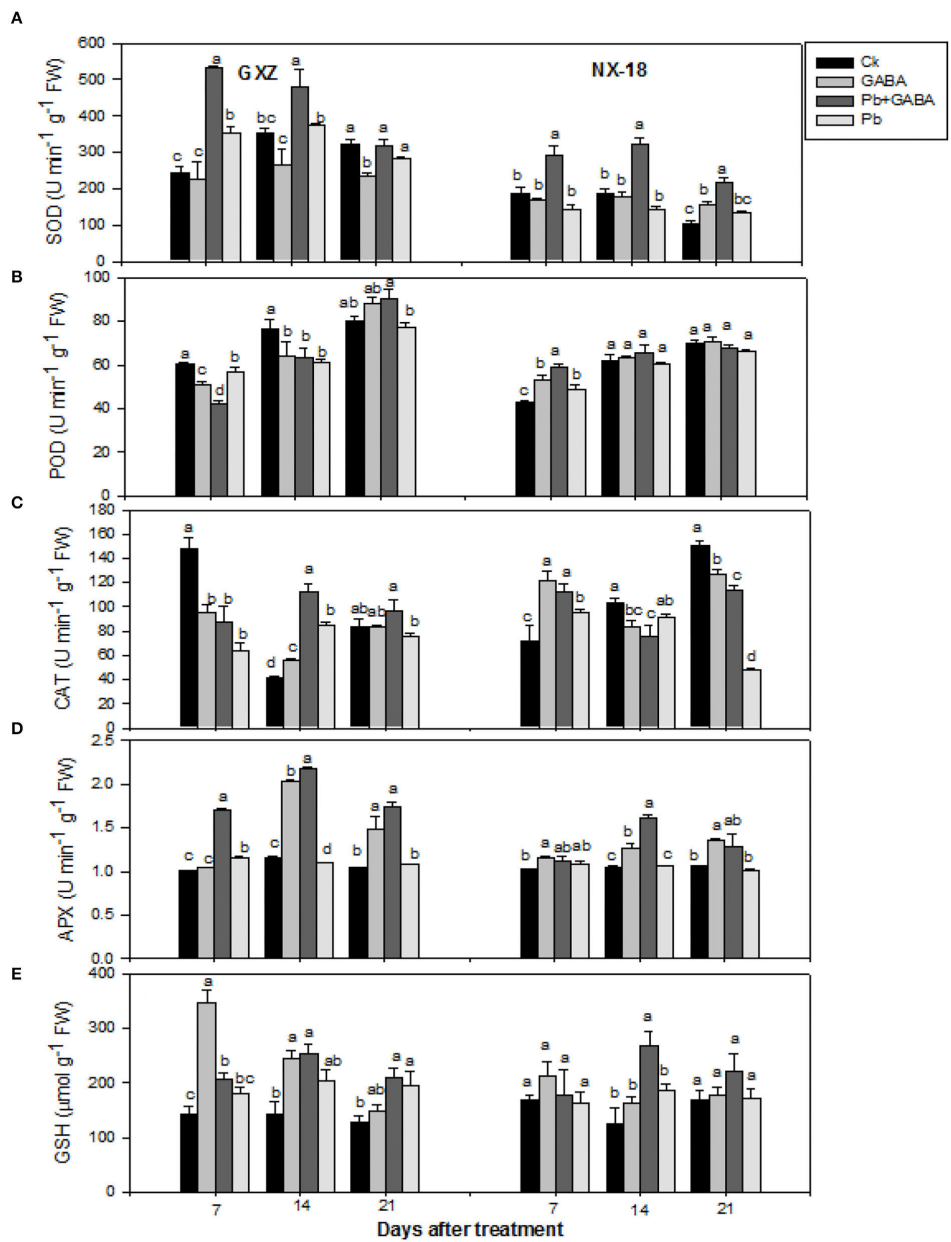
Influence of exogenous GABA on **(A)** superoxide dismutase (SOD), **(B)** peroxidase (POD), **(C)** catalase (CAT), **(D)** ascorbate peroxidase (APX) activities, and **(E)** reduced glutathione (GSH) contents in fragrant rice under Pb toxicity at 7, 14, and 21 days after treatment. Vertical bars are means ± S.E. Vertical bars sharing a letter in common do not differ significantly at *P* < 0.05. Ck: without Pb and GABA (control); GABA: 1 mM GABA; Pb + GABA: 1 mM GABA + 800 mg of Pb kg^−1^ of soil; Pb = 800 mg kg^−1^ of soil. GXZ, Guixiangzhan; NX-18, Nongxiang-18.

Exogenous GABA application modulated the antioxidant activities, that is, SOD, POD, CAT, APX, and GSH contents significantly under Pb toxicity. For instance, SOD activities were found the highest in Pb + GABA treatment at all sampling intervals in both GXZ and NX-18, while significantly higher POD activities were recorded in Ck at 7 and 14 DAT and in Pb + GABA at 21 DAT in GXZ. In NX-18, except at 7 DAT, where POD activity was significantly higher in Pb + GABA than in other treatments, the POD activity remained statistically similar (*P* > 0.05) for all the treatments at 14 and 21 DAT. Furthermore, in GXZ, the CAT and APX activities were substantially higher in plants under Pb + GABA at all sampling stages (except CAT at 7 DAT, where the highest CAT activity was recorded in Ck). In NX-18, reduced CAT activities were recorded in Pb-exposed plants even with GABA application except for CAT at 7 DAT (maximum CAT activity under GABA-treated plants) than in Ck. The APX activities were found higher in Pb-exposed plants even with or without GABA treatment than Ck at 7, 14, and 21 DAT; however, the activity of APX in Ck was marginally higher than APX activity in Pb treatment at 21 DAT. Likewise, GABA promoted the GSH contents in both rice cultivars under Pb stress. At 7 DAT, the maximum GSH contents were recorded in GABA-applied plants without Pb, while at 14 and 21 DAT, the maximum GABA contents were found in the Pb + GABA treatment. Furthermore, GABA application also enhanced GSH contents in NX-18 but the effects remained only significant at 14 DAT, whereas, at 7 and 21 DAT, the GSH contents remained statistically similar (*P* > 0.05) for all the treatments (Figure 5).

### GS and NR Activities

**Figure 6:**
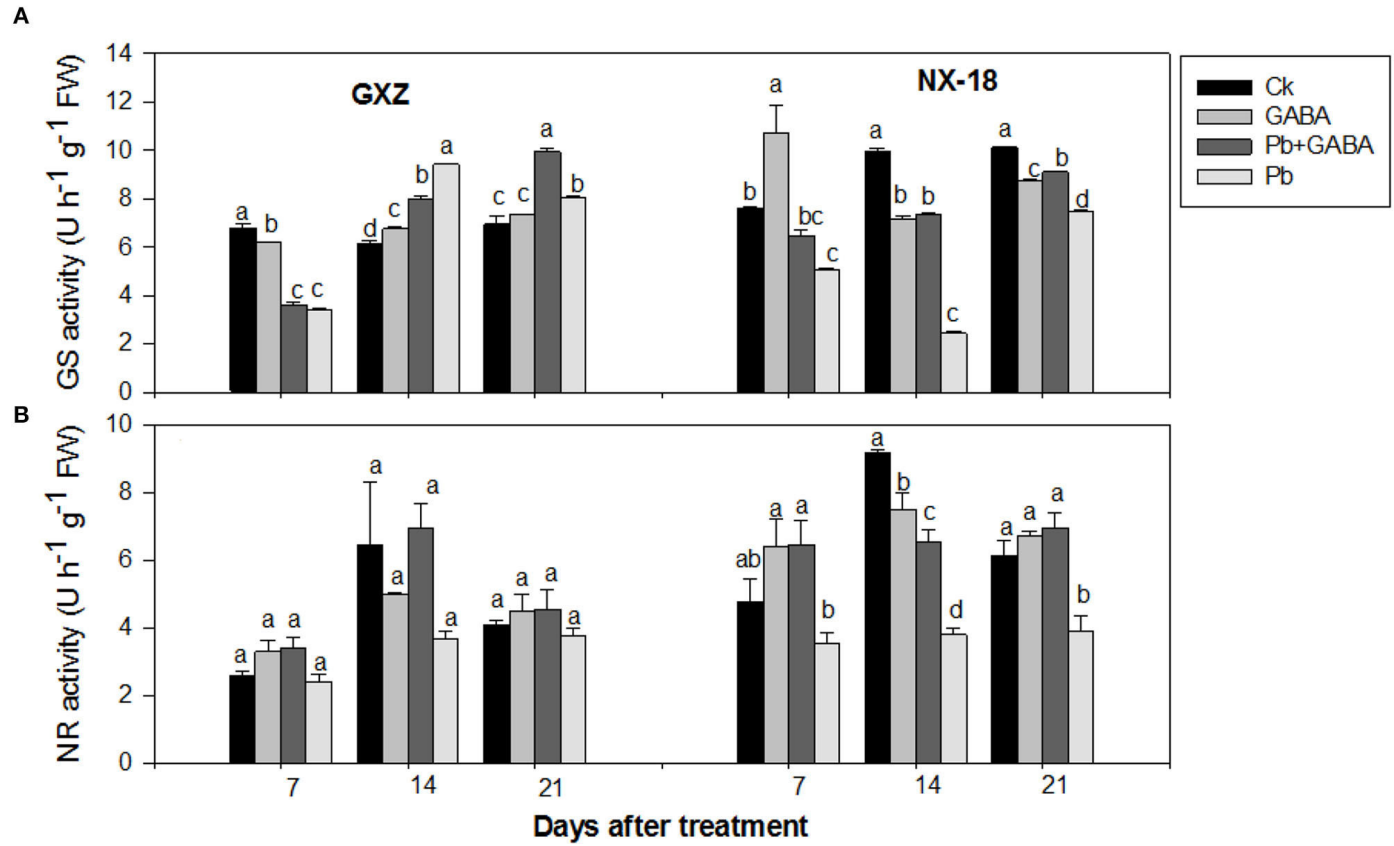
Influence of exogenous GABA on **(A)** glutamine synthetase (GS) and **(B)** nitrate reductase (NR) activities in fragrant rice under Pb toxicity at 7, 14, and 21 days after treatment. Vertical bars are means±S.E. Vertical bars sharing a letter in common do not differ significantly at *P* < 0.05. Ck: without Pb and GABA (control); GABA: 1 mM GABA; Pb + GABA: 1 mM GABA + 800 mg of Pb kg^−1^ of soil; Pb = 800 mg kg^−1^ of soil. GXZ, Guixiangzhan; NX-18, Nongxiang-18.

Both GS and NR activities were variably affected due to GABA application in both rice cultivars under Pb stress. For example, in GXZ, the highest GS activities were recorded in Ck, Pb, and Pb + GABA treatments at 7, 14, and 21 DAT, respectively, while NR activity was found higher in GABA-treated plants (with or without Pb) but statistically similar (*P* > 0.05) for all the treatments at all sampling stages. On the other hand, for NX-18, the maximum GS activity was recorded in GABA treatment at 7 DAT, while in Ck at 14 and 21 DAT, but the lowest in Pb-treatment without GABA application. Similarly, Pb reduced the NR activity by 25.79, 58.58, and 36.33% in NX-18 than in Ck, while the highest NR activity was recorded in Pb + GABA treatment at 7 and 21 DAT, and in Ck at 14 DAT. In general, the reduction in GS and NR activities was more severe in NX-18 than GXZ under Pb stress (Figure 6).

### Pb Contents in Different Plant Parts

Both rice cultivars showed differential behavior regarding Pb accumulation in different plant parts; however, the highest Pb contents were recorded in roots followed by stems, leaves (at VEG), ears (at PH), and grains (at MAT) stages in both GXZ and NX-18. Exogenous application of GABA in plants under Pb toxicity significantly reduced Pb contents in different plant parts compared with Pb exposed plants without GABA. For example, at the VEG stage, the Pb contents 27.37 and 29.70% (in roots), 16.39 and 21.70% (in stems), and 62.50 and 35.01% (in leaves) were lower in GABA + Pb than only in Pb-exposed plants of GXZ and NX-18, respectively. Similarly, the Pb contents in roots, stems, leaves, and ears under Pb + GABA-treated plants at the PH stage were 36.07 and 34.15%, 53.50 and 32.30%, 113.58 and 84.98%, and 60.01 and 58.81% lower in Pb + GABA for GXZ and NX-18, respectively, compared with Pb treatment, while 25.65 and 26.58% lower Pb contents in roots, 18.18 and 23.36% in stems, 34.04 and 50.94% in leaves, and 67.06 and 38.38% in grains were recorded in GXZ and NX-18, respectively, under Pb + GABA than only in Pb-exposed plants. Overall, Pb + GABA showed substantially lower Pb accumulation in both rice cultivars than Pb-exposed plants without GABA application (Table 1).

**Table 1:** Influence of exogenous gamma-amino butyric acid (GABA) application on the accumulation of Pb contents (μg g^−1^) in different plant parts of fragrant rice.

**Cultivars**	**Treatments**	**Tillering**			**Panicle heading**				**Maturity**			
		**Root**	**Stems**	**Leaves**	**Root**	**Stems**	**Leaves**	**Ears**	**Root**	**Stems**	**Leaves**	**Grains**
**GXZ**	Ck	28.99 c	6.02 c	4.97 c	23.40 c	6.36 c	5.58 c	0.80 c	25.02 c	4.96 c	2.97 c	0.20 c
	GABA	25.69 c	6.19 c	3.29 c	26.00 c	8.05 c	5.03 c	0.63 c	26.52 c	6.10 c	3.38 c	0.16 c
	Pb + GABA	1,547.94 b	98.26 b	21.40 b	1,758.70 b	85.10 b	29.00 b	8.05 b	2,075.13 b	106.31 b	75.71 b	1.96 b
	Pb	1,971.64 a	114.37 a	34.77 a	2,393.11 a	130.63 a	61.93 a	12.89 a	2,607.47 a	125.64 a	101.48 a	3.27 a
	LSD_0.05_	164.49	4.78	5.97	171.18	9.16	6.82	3.42	116.88	4.51	8.58	0.58
**NX-18**	Ck	17.25 c	6.60 c	4.54 c	18.63 c	6.80 c	5.78 c	0.97 c	22.33 c	5.26 c	5.49 c	0.51 c
	GABA	18.46 c	7.25 c	4.35 c	16.40 c	8.05 c	4.97 c	0.72 c	21.10 c	6.29 c	5.02 c	0.40 c
	Pb + GABA	1,616.88 b	111.15 b	38.98 b	1,853.22 b	116.11 b	42.89 b	17.93 b	1,906.83 b	128.07 b	85.37 b	6.43 b
	Pb	2,097.16 a	135.31 a	52.63 a	2,486.02 a	153.61 a	79.33 a	28.47 a	2,413.66 a	157.98 a	128.87 a	8.90 a
	LSD_0.05_	202.24	4.97	6.20	171.18	11.01	5.22	2.14	203.10	6.68	4.81	1.50

*Values are the means of three replicates S.E. Values sharing a letter in common within the same column do not differ significantly at the 5% probability level according to the least significant difference (LSD) test. Ck: without Pb (control), GABA: 1 mM GABA, Pb + GABA: 1 mM GABA + 800 mg of Pb kg^−1^ of soil, Pb = 800 mg kg^−1^ of soil. GXZ, Guixiangzhan; NX-18, Nongxiang-18*.

### Yield and Related Components

Yield and related traits of both rice cultivars were severely affected by Pb toxicity but some of the yield-related traits, that is, filled grain % and 1,000 grain weight (in GXZ) and grains per panicle (in NX-18) were remained statistically at par (*P* > 0.05) for all the treatments. However, exogenous GABA application improved yield and yield components considerably as the percentage reduction under Pb-exposed plants (without GABA) was higher than only GABA-applied plants. For example, Pb treatment led to a reduction in productive tillers/pot (13.64 and 10.29%), grains/panicle (0.37 and 2.26%), filled grain % (3.89 and 19.06%), 1,000-grain weight (7.35 and 12.84%), and grain yield (17.92 and 40.56) as compared with Pb + GABA. Overall, the yield reduction was more severe in NX-18 than GXZ, while the highest yield and yield components were recorded in plants only under GABA application without Pb (Table 2).

**Table 2:** Influence of exogenous gamma-amino butyric acid (GABA) application on yield and related components of fragrant rice under Pb toxicity.

**Rice cultivars**	**Treatments**	**Productive tillers/pot**	**Grains/panicle**	**Filled grain %**	**1000-grain weight (g)**	**grain yield/pot (g)**
**GXZ**	Ck	30.41 ± 0.41 a	167.17 ± 1.69 ab	79.45 ± 3.75 a	20.05 ± 0.36 a	69.54 ± 3.68 ab
	GABA	31.53 ± 0.87 a	176.90 ± 3.55 a	78.14 ± 2.95 a	20.04 ± 0.17 a	75.58 ± 3.41 a
	Pb + GABA	26.63 ± 0.86 b	163.87 ± 2.19 b	72.76 ± 0.21 a	19.82 ± 0.10 a	62.34 ± 1.31 b
	Pb	23.00 ± 1.15 c	163.27 ± 6.14 b	69.93 ± 5.19 a	18.37 ± 0.38 a	51.17 ± 2.03 c
	Means	27.89	167.80	72.27 3.89	19.57	64.66
	LSD_0.05_	3.17	12.55	10.64	1.08	10.49
**NX-18**	Ck	26.83 ± 1.17 a	162.80 ± 6.40 a	81.87 ± 5.73 a	20.02 ± 1.03 a	57.46 ± 2.12 b
	GABA	27.48 ± 1.06 a	151.20 ± 1.94 a	81.82 ± 1.35 a	20.48 ± 0.35 a	63.39 ± 1.68 a
	Pb + GABA	22.67 ± 1.45 b	147.33 ± 5.21 a	70.83 ± 1.89 ab	19.96 ± 1.13 a	48.28 ± 2.71 c
	Pb	20.33 ± 1.45 b	144.00 ± 6.66 a	57.33 ± 5.58 b	17.40 ± 0.60 b	28.70 ± 0.40 d
	Means	24.33	151.33	75.32	19.47	49.46
	LSD_0.05_	3.91	20.89	15.94	1.36	4.46

*Ck: without Pb (control); GABA: 1 mM l^−1^ GABA; Pb + GABA: 1 mM GABA + 800 mg of Pb kg^−1^ of soil; Pb = 800 mg kg^−1^ of soil. GXZ, Guixiangzhan; NX-18, Nongxiang-18*.

## Discussion

Exogenous GABA-induced regulations on physiological, biochemical, and yield of two fragrant rice cultivars were assessed in this study. It was found that Pb significantly elevated the lipid peroxidation, electrolyte leakage, and H_2_O_2_ contents, while the exogenous GABA application reduced the oxidative stress in both rice cultivars. Comparatively, the degree of oxidative damage was higher in NX-18 than in GXZ even with or without GABA (Figure 1). Plants produced reactive oxygen species (ROS) as signaling molecules for any kind of stress; however, under acute stress conditions, the rate of ROS production was accelerated enough that ROS starts to oxidize and/or degrade essential cellular structures and distort cell normal functionality. Enhanced MDA contents showed that lipid peroxidation and electrolyte leakage are the loss of membrane integrity, whereas H_2_O_2_ is a potential ROS (at higher levels) that reacts with micro and macro biological molecules and disturbs their structures and functions. Meanwhile, plants treated with GABA showed comparatively less oxidative stress than non-treated plants, which depicts that GABA plays some role in scavenging ROS to reduce oxidative damage in fragrant rice under Pb toxicity, whereas the high oxidative stress in NX-18 than GXZ showed that NX-18 is sensitive to Pb stress. The GABA may have some ROS scavenging and/or regulatory roles in plants (Shi et al., 2010), as the GABA shunt pathway provides succinate and nicotinamide adenine dinucleotide (NADH) during respiration, hence regulations in GABA might have a direct role in regulating the ROS/RO intermediates under oxidative stress (Bouche and Fromm, 2004). The reduced oxidative stress in GABA-treated plants under metal toxicity was also reported previously (Nayyar et al., 2014; Kumar et al., 2019). Generally, the GABA rapidly accumulates in plants under abiotic stress conditions, thus regulations in the endogenous GABA levels substantially affect the plant morphogenesis. The GABA-induced stress tolerance is associated with the expression of genes responsible for the biosynthesis of phytohormones, plant signaling, redox reactions, transcriptional regulation, and polyamine metabolism. Hence, associations of GABA with polyamines and phytohormones could improve the plant performance under stress conditions (Podlešáková et al., 2019).

The GABA also improved chlorophyll and carotenoids (Figure 2), proline, protein, and GABA contents (Figure 3), net photosynthesis, and gas exchange attributes (Figure 4), as well as modulating the antioxidative activities (Figure 5) in fragrant rice under Pb toxicity, whereas such improvements were prominent in GXZ than NX-18. The reduction in the leaf chlorophyll contents is the distinctive character of Pb-induced oxidative stress as a consequence of chlorophyll degradation due to enhanced chlorophyllase activity and/or photo-oxidation (Hegedus et al., 2001) of ROS. This reduction in chlorophyll contents might lead to reduced photosynthesis and gas exchange in both rice cultivars. The GABA provided partial protection to the photosynthetic pigments, by scavenging the degenerative ROS activities *via* proline accumulation and/or activation of the antioxidative defense system (Khanna et al., 2021). These inter-linked physiological mechanisms are modulated by GABA to improve rice performance under Pb stress. The GABA-induced modulations in photosynthetic machinery, especially “photosystem I and II,” were noted in *Piper nigrum* Linn (Vijayakumari and Puthur, 2016). Alternatively, non-significant improvements due to GABA application were noted in chlorophyll contents and chlorophyll fluorescence, except for photochemical quenching coefficient, and actual photochemical efficiency in hydroponically cultured tomato seedlings under salt stress (Luo et al., 2011). In addition to cytosolic adjustments, proline is involved actively in scavenging ROS and protecting proteins and thylakoid membrane under stress conditions (Hayat et al., 2012). The Pb stress led to a significant increase in proline contents in aromatic rice cultivars (Ashraf et al., 2017b), whereas Li W. et al. (2016) reported substantial enhancements in proline contents in maize under GABA application. GABA application promoted SOD, POD, CAT, and APX activities either in Pb stressed and non-stressed rice plants or in Pb stress only (without GABA). Both the SOD and CAT play a significant role in cleansing ROS; for example, SOD detoxifies super-oxide anion to O_2_ and then H_2_O_2_, which, later, is further reduced by CAT and POD enzymes (Alscher et al., 2002). The APX and GSH are involved in the ascorbate-glutathione cycle that detoxifies the harmful effects of ROS (Ali et al., 2014a). The lower antioxidant activities in plants under Pb stress without GABA showed that the production rate of ROS may be too high to be scavenged by antioxidants activities and/or reduced ROS scavenging efficiencies; however, exogenous application of GABA improved the antioxidant activities by lowering ROS production and enhancing scavenging abilities of antioxidants. Gupta et al. (2010) also stated that antioxidants have a principal role to quench the Pb-caused production of ROS. GABA-induced maintenance in energy status and enhanced chilling tolerance due to modulations in enzymatic and non-enzymatic antioxidants were previously noted in peach fruit under chilling stress (Yang et al., 2011). Our findings are further concomitant with Shi et al. (2010) who found significant modifications in antioxidants to remove ROS in *Caragana intermedia* plants due to GABA application. Pb stress reduced the GS and NR activity in both GXZ and NX-18; however, their activities were found higher in GABA-treated plants than in non-GABA under Pb stress. The reduction in GS and NR activities was comparatively higher in NX-18 than in GXZ (Figure 6). Generally, GABA contributes to maintaining the C-N equilibrium by regulating N-metabolism and/or by controlling the transport and storage of nitrogen within plant organs. The GABA shunt pathway also regulates C:N fluxes by actively assimilating the glutamate-generated carbons (Bouche and Fromm, 2004). Positive correlations were also observed between NR activity and nitrate influx (higher nitrate uptake) in growing rape plants (Beuve et al., 2004). Regulations in the activities of NR and GS were also observed in *Arabidopsis thaliana* seedlings under GABA application (Barbosa et al., 2010). The GABA application improved the Cd tolerance in maize by upregulating the antioxidant enzyme activities and polyamine biosynthesis-responsive genes, that is ornithine decarboxylase and spermidine synthase (Seifikalhor et al., 2020). Hence, GABA helped rice plants to maintain GS and NR activity under Pb stress, while, comparative to GXZ, reduced GS and NR activities in NX-18, depicting that Pb toxicity badly affected the C-N metabolism in this cultivar. Previously, modulation in GS and NR activities in maize (Li M. et al., 2016; Li W. et al., 2016) was noted due to GABA, which shows the involvement of GABA in C:N metabolism; however, the mechanism and extent of its involvement in C-N metabolism are still not clear.

Exogenous GABA application improved the yield and related components, while it reduced Pb uptake and accumulation in stems, leaves, and grains under Pb stress. The yield loss was more severe in NX-18 (with more Pb contents in upper plant parts) than in GXZ (Tables 1, 2). Reductions in yield-related components might lead to reduced grain yield in both rice cultivars. Previously, Liu et al. (2003) declared that relative changes in spikelets per panicle under Pb toxicity have significant and positive correlations with the relative changes in rice grain yield, whereas effects of Pb toxicity were more severe for spikelets per panicle and rice grain weight. In general, relative changes in rice grain yield were related to productive tillers, grain per panicle, and 1,000-grain weight under Pb toxic conditions (Ashraf et al., 2020). Improve chlorophyll contents, net photosynthesis, proline accumulation, antioxidative defense mechanism, and balanced GS and NR activity due to GABA application possibly led to improved yield and yield components in both GXZ and NX-18; however, the improvements were more prominent in GXZ that might be due to its tolerant behavior against Pb. GABA application-induced improvements in yield and quality attributes of fragrant rice were also reported by (Xie et al., 2020).

**Figure 7:**
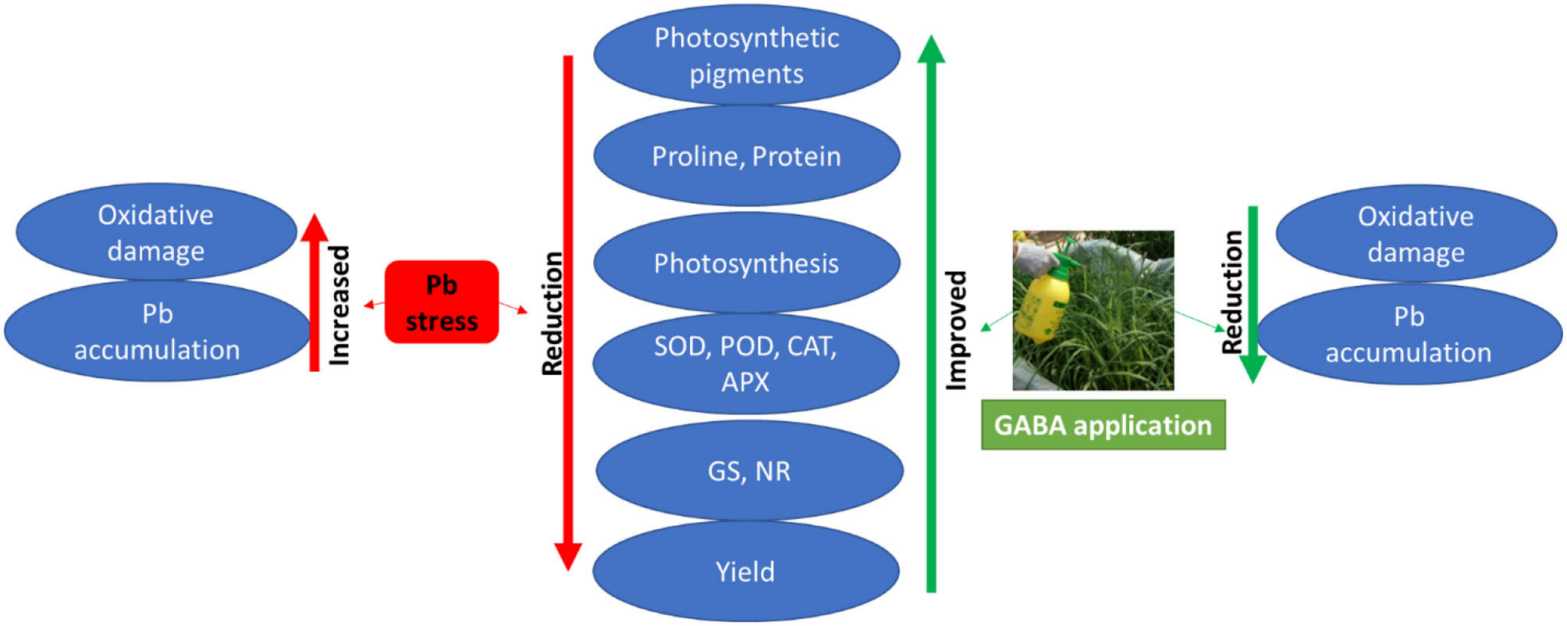
Effects of exogenous GABA application on physio-biochemical attributes and yield as well as Pb accumulation in fragrant rice under Pb stress.

In addition, Pb uptake in plants is largely affected by different plant and soil factors (Bharwana et al., 2014); however, less Pb contents in GABA-treated plants may be due to the protective role of GABA and maintenance of cell integrity, thus less Pb intake into the plants. Moreover, the signaling role of GABA could also help plants to respond effectively against Pb stress. Ramesh et al. (2015) found that GABA-induced Al tolerance was due to aluminum-activated malate transporters (ALMT) in wheat; nevertheless, the exact mechanism of GABA involvement to reduce Pb contents in upper plant parts and molecular basis of Pb tolerance due to GABA application are yet to be explored. Overall, the positive effects of exogenous GABA application on rice under Pb stress have been summarized in (Figure 7).

## Conclusion

In conclusion, Pb toxicity adversely affected the physiology and grain productivity of both rice cultivars; however, exogenous GABA application reduced oxidative damage, improved chlorophyll contents and photosynthesis, and regulated the antioxidant defense mechanism as well as GS and NR in both rice cultivars. The GABA application improved yield and related components in both GXZ and NX-18 under Pb stress; nevertheless, the yield reduction was higher in NX-18 than in GXZ. The Pb contents in different plant parts were recorded as root > stems > leaves > ears (at panicle heading) > grains (at maturity); nonetheless, the concentrations were substantially lower in respective organs under GABA treatments. Hence, rather than just acting as a signaling molecule, the GABA may also be applied exogenously as an “osmolyte” to induce Pb stress tolerance in rice due to its multi-functional role.

## Data Availability Statement

The original contributions presented in the study are included in the article/supplementary material, further inquiries can be directed to the corresponding authors.

## Author Contributions

UA and XT designed the experiment. UA and ZM investigated the traits and lab analyses. UA, SM, and SAA analyzed the data and wrote the manuscript. FR, RNA, JI, and XT revised and edited the manuscript. All authors read and approved the final version of the manuscript. All authors contributed to the article and approved the submitted version.

## Funding

This study was supported by the National Natural Science Foundation of China (31271646), the Natural Science Foundation of Guangdong Province (8151064201000017) China, and the China Scholarship Council (CSC), China.

## Conflict of Interest

The authors declare that the research was conducted in the absence of any commercial or financial relationships that could be construed as a potential conflict of interest.

## Publisher's Note

All claims expressed in this article are solely those of the authors and do not necessarily represent those of their affiliated organizations, or those of the publisher, the editors and the reviewers. Any product that may be evaluated in this article, or claim that may be made by its manufacturer, is not guaranteed or endorsed by the publisher.
